# In the COVID-19 pandemic, who did we trust? An eight-country cross-sectional study

**DOI:** 10.7189/jogh.13.06036

**Published:** 2023-09-01

**Authors:** Alexa P Schluter, Mélissa Généreux, Elsa Landaverde, Philip J Schluter

**Affiliations:** 1 Mātai Tōrangapū | Department of Political Science and International Relations, Te Whare Wānanga o Waitaha | University of Canterbury, Ōtautahi | Christchurch, Aotearoa | New Zealand; 2 Department of Community Health Sciences, Faculté de Médecine et des Sciences de la Santé, Université de Sherbrooke, Sherbrooke, Canada; 3 Department of Community Health Sciences, Faculté de Médecine et des Sciences de la Santé, Université de Sherbrooke, Sherbrooke, Canada; 4 Te Kaupeka Oranga | Faculty of Health, Te Whare Wānanga o Waitaha | University of Canterbury, Ōtautahi | Christchurch, Aotearoa | New Zealand; 5 School of Clinical Medicine, Primary Care Clinical Unit, The University of Queensland, Brisbane, Australia

## Abstract

**Background:**

Trust is a key determinant of health, but has been undermined by the COVID-19 pandemic and the associated infodemic. Using data from eight countries, we aimed to epidemiologically describe levels of trust in health, governments, news media organisations, and experts, and measure the impact of political orientation and COVID-19 information sources on participant’s levels of trust.

**Methods:**

We simultaneously conducted a stratified randomised online cross-sectional study across eight countries on adults aged ≥18 years between 6 and 18 November 2020. We employed crude and adjusted weighted regression analyses.

**Results:**

We included 9027 adults with a mean age of 47 years (range = 18-99), of whom 4667 (51.7%) were female. Trust in health experts ranked highest across all countries (mean (x̄) = 7.83; 95% confidence interval (CI) = 7.79-7.88), while trust in politicians ranked lowest (x̄ = 5.34; 95% CI = 5.28, 5.40). In adjusted analyses, political orientation and utilised information sources were significantly associated with trust. Individuals using higher levels of health information sources trusted health authorities more than those using lower levels (mean difference = 1.12; 95% CI = 1.02, 1.14). Similarly, individuals using higher levels of government information sources (mean difference = 1.55; 95% CI = 1.43, 1.64) and those using higher levels of new media information sources (mean difference = 1.17; 95% CI = 1.06, 1.28) had highest trust in governments/politicians and news media, respectively. However, there was little difference in trust in health, government, or news media between individuals using higher or lower levels of social media information sources.

**Conclusions:**

Trust is a key determinant of health, but has been politically fragile during this infodemic. High compliance with public health measures is key to combatting infectious diseases. In terms of people’s trust, our findings suggest that politicians and governments worldwide should coordinate their response with health experts and authorities to maximise the success of public health measures.

With a conservatively estimated 6.95 million attributable deaths reported to the World Health Organization (WHO) [1], the coronavirus disease 2019 (COVID-19) continues to profoundly impact global health and economy [2]. Many governments initially pursued restrictive elimination strategies to reduce COVID-19 community transmission and minimise the strain on their health systems [3,4]. Promoting and sustaining population compliance with public health measures was invariably crucial to these strategies for reducing preventable mortality and morbidity.

People’s trust is a key component for population compliance. It directly impacts people’s risk perception, social cohesion, and behavioural changes and has been recognised as foundational in determining a country’s mortality and morbidity trajectory [5-7]. Countries which were the most successful in curbing COVID-19 infections were not those with the best health care systems or experts, but rather those with high levels of national and community trust [8].

Trust can take several forms, ranging from an individual’s trust in national governments and officials to one’s interpersonal trust in their surrounding community. A 177-country study investigating pandemic preparedness found significantly lower standardised infection rates with higher government and interpersonal trust [8]. While associative rather than causative, this finding suggests that increasing trust and compliance may reduce the global infection burden. Although some factors are largely immutable (such as demographics and colonisation histories), leaders and media influence levels of trust. However, it has been argued that the increasing politicization of public health is undermining trust in health care systems across the globe [9]. This has led to persistent inequalities of access and outcomes [8] fuelled by a widespread infodemic [10], resulting in trust being asserted as a key determinant of health [9].

Political orientation and its relationship to trust have been found to significantly impact COVID-19 prevention behaviours [11-13]. The ideologies underlying political identities may affect a person’s belief in their national governments and institutions, particularly if they disagree with the current party in office [11]. A study conducted in Italy found that the degree of compliance to social distancing orders was higher in provinces with greater political support for current legislation. In provinces with high opposition support, individuals mostly did not adhere to social distancing orders [11]. Understanding how factors such as political alignment may impact an individual’s trust is vital in the development of policy aimed at enhancing compliance for recommended guidelines and health mandates.

The WHO identified the “infodemic”, referring to the rapid spread of both accurate and inaccurate information in the age of the internet and social media, as a key challenge in responding to COVID-19 and other disease outbreaks [14]. Misinformation and disinformation can provide simple, yet erroneous explanations to help make frightening situations more comprehensible [15]. There are many examples where misinformation and disinformation campaigns and conspiracy theories have minimised the threat of the virus itself and negatively impacted the overall health of a society and its social cohesion, resulting in lives lost [16,17]. Understanding and managing the infodemic is thus a matter of public health importance, and investigating how trust is shaped is key to understanding information sources.

Using an eight-country cross-sectional study design, which recruited representative samples of adults, we aimed to investigate people’s level of trust and how it was affected by their political orientation and information sources. Our primary goals were to epidemiologically describe levels of trust in health, government, and news media organisations and experts and measure the impact of participant’s political orientation and COVID-19 information sources on their levels of trust.

## METHODS

### Study design

We conducted a stratified randomised online cross-sectional study in seven countries (Canada, United States of America (USA), England, Switzerland, Belgium, Philippines, New Zealand) and one territory (Hong Kong) simultaneously between 6 and 18 November 2020. As Hong Kong is a special administrative region of China and enjoys governing and economic autonomy, we referred to it as a country rather than a territory. The timing of this study coincided with the beginning of the second COVID-19 wave [1], when the disease was still potentially severe, restrictive measures were re-implemented, no vaccines were available, rising in pandemic fatigue was observed [18], and when the divisive presidential election were ongoing in the USA.

### Participants

We included adults aged ≥18 years who resided of participating countries at the time of the survey.

### Primary outcome measures

Informed by the Organisation for Economic Co-operation and Development (OECD) guidelines [19], we designed six questions covering different aspects of trust:

1. In scientists, doctors, and health experts;
2. In national health organisations;
3. In global health organisations;
4. In government;
5. In politicians;
6. In news organisations.

Response options for each used a 10-point scale, ranging from 1 (very low) to 10 (very high) levels of trust.

## Primary explanatory measures

We asked participants about their political orientation via the question “When it comes to politics, people talk about ‘left’ and ‘right’. Where would you place yourself on a scale, where 0 stands for far left and 6 for far right?”. Due to the political sensitivities in Hong Kong preceding instrument development [20], we did not question Hong Kong participants on their political orientation.

Regarding information sources, the participants were asked “To what extent do you use the following sources to inform yourself about the coronavirus (also known as COVID-19)?”. We devised 11 possible responses:

1. The federal government;
2. Provincial government;
3. Politicians;
4. World Health Organization (WHO);
5. Health professionals in the media;
6. Public health authorities;
7. Television;
8. Radio;
9. Newspapers (including online);
10. Social networks (Facebook, Twitter, Instagram, etc.);
11. Other internet sources.

Each question had additional response options which ranged from 1 to 4 (1 = mainly/always, 2 = often, 3 = sometimes, and 4 = not much/never). We collapsed questions into four domains: health (questions 4-6), government (questions 1-3), news media (questions 7-9), and social media (questions 10-11). We calculated summed response scores for each domain and standardised them by dividing by the number of contributing variables. We dichotomised these re-scaled scores into higher (score ≤2.5) and lower (score >2.5) categories, with this 2.5 threshold representing the mid-point on the four-point response option scale, falling between “often” and “sometimes”.

### Sociodemographic and potentially confounding variables

A detailed account of the sociodemographic variables is presented elsewhere [21]. In brief, participants could identify their gender as “Male”, “Female”, “Another gender identity”, and “I don’t know/I prefer not to answer”. Due to the few gender-diverse respondents and those answering “I don’t know/I prefer not to answer”, we set these genders to missing to allow for meaningful statistical analysis. We categorised age groups as 18-24, 25-34, 35-44, 45-54, 55-64, 65-74, and ≥75 years of age. We categorised usual household composition as living alone, with spouse/partner only, with spouse/partner and child(ren), other family (including single parent), and other non-family arrangements. We asked participants if they were an essential worker (e.g. health care and social services, law enforcement, emergency services, provider of essential goods, educational institution) with response options being “Yes”, “No”, and “I don’t know/I prefer not to answer”. Those who responded affirmatively were asked in which essential sector they usually worked. Participants who worked in health care and social services were further partitioned from the other essential workers. The perceived threat that COVID-19 posed to self and country were separately assessed on a five-point scale, with response options being “Very low”, “Low”, “Moderate”, “High”, and “Very high”. We also queried the participants on financial losses linked to COVID-19 up to the time of the survey, with response options being “None”, “Insignificant”, “Significant”, “Very Significant”, and “I don’t know/prefer not to answer”. Finally, we examined stress by asking the question: “Thinking about the level of stress in your life, would you say that most of your days are”, with response options being “Not stressful at all”, “Not very stressful”, “A bit stressful”, “Rather stressful”, and “Extremely stressful”.

### Procedure

A detailed description of the procedure is available elsewhere [21,22]. Country selection was governed by practical considerations, including ensuring global continent and COVID-19 burden and response diversity within a constrained budget and timeframe, together with having country-specific lead investigators to provide linguistically and culturally appropriate insight and context. The core team came together from pre-existing professional connections, including the WHO Thematic Platform for Health Emergency and Disaster Risk Management Research Network. Due to time urgency and funding constraints, this core team purposely approached and invited potential research leads in identified countries [21,22]. Once finalised, the international research team and partners then developed the survey instrument, checking and confirming its language and content suitability for each participating country and making it available in English, French, German, Italian, and Chinese languages. Prior to the full eight country study, the instrument was successfully piloted within Canada [21]. Two polling firms, in collaboration with international partners, then undertook random participant recruitment and data collection using an online platform. The sampling frame included online and offline sources, encompassing, where available, publicly available records (such as electoral roles). To ensure recruitment and representation of hard-to-reach sub-populations, quota sampling was employed and tailored for each country based on the latest available population census; it comprised of age, gender, and region stratifications. After contact, eligibility confirmation, and informed consent, participants completed the survey which took approximately 20 minutes.

We set a minimum sample size of 1000 adults for each participating country, except for Canada (the research program host), which we set at 2000. This was to obtain a largely balanced sample sizes for each country, thereby maximising statistical power for intercountry comparisons, to gain reasonable intra-country power (detecting differences in proportions of ≥10% or a relative risk of ≥1.2 exceeding 80% for a two-tailed α = 0.05), and to maximise country inclusion and diversity within a constrained budget and timeframe.

### Statistical analysis

We used the STrengthening the Reporting of OBservational studies in Epidemiology (STROBE) guidelines in reporting this study [23]. We assigned survey sampling weights to participant data, calibrated to match population census distributions, correcting for any unequal representation due to quotas not being fully achieved. We conducted all analyses using Stata SE version 17.0 (StataCorp, College Station, USA), accommodated survey sampling weights, and employed robust variance estimators. We considered a two-tailed α = 0.05 as denoting significance.

We initially described and compared participant numbers and sociodemographic characteristics by countries using Pearson design-based F-test. We assessed correlation between primary variables using weighted Pearson pairwise correlation coefficients (*R*). Next, we conducted crude and adjusted complete case regression analyses relating participants’ political orientation and information source level to their trust in health authorities, government/politicians. Rather than employing bivariable analyses to screen sociodemographic and potential confounding factors, as suggested by Sun et al. [24], we included all variables in the adjusted model regardless of their significance. We reported means (x̄) and associated 95% confidence intervals (CIs), and used Wald’s type III χ^2^ statistic to determine the significance of variables within the regression models.

## RESULTS

### Participants and their characteristics

We included 9027 adults with a mean age of 47 years (standard deviation (SD) = 17.0 years), with the oldest participant being 99 years; 4667 (51.7%) were female, 4318 (47.8%) male, 28 (0.3%) neither female nor male, and 14 (0.2%) preferred not to answer the question. Regarding household composition, 2680 (29.7%) participants lived with their spouse/partner and 2069 (22.9%) resided with their spouse/partner and child(ren). The majority (n = 6450 (73.3%)) were non-essential workers, although 800 (9.1%) participants reported being essential workers in health (**Table 1**).

**Table 1:** Demographic characteristics of participants by country

	Canada	USA	England	Belgium	Switzerland	Hong Kong	Philippines	NZ
**Age in years, mean (SD)**	47.8 (17.2)	48.4 (17.2)	47.6 (17.1)	49.4 (16.2)	50.1 (17.1)	46.5 (15.7)	38.1 (14.7)	46.9 (17.0)
Gender, n (%)*								
Female	1031 (51.7)	517 (51.9)	511 (51.2)	520 (51.6)	522 (52.2)	550 (55.0)	503 (50.7)	513 (51.4)
Male	963 (48.3)	478 (48.1)	487 (48.8)	489 (48.4)	477 (47.8)	451 (45.0)	489 (49.3)	484 (48.6)
**Usual household composition, n (%)**								
Alone	366 (18.3)	230 (22.9)	212 (21.2)	191 (18.8)	271 (27.1)	67 (6.7)	34 (3.4)	156 (15.6)
Partner only	743 (37.1)	267 (26.6)	333 (33.3)	389 (38.3)	373 (37.3)	179 (17.9)	104 (10.3)	293 (29.3)
Partner & child(ren)	428 (21.4)	224 (22.3)	228 (22.8)	232 (22.9)	177 (17.7)	311 (31.1)	203 (20.2)	265 (26.5)
Other family†	388 (19.4)	251 (25.0)	201 (20.1)	185 (18.3)	150 (15.0)	435 (43.4)	636 (63.4)	242 (24.1)
Non-family‡	79 (3.9)	31 (3.1)	26 (2.6)	17 (1.7)	30 (3.0)	10 (1.0)	26 (2.6)	46 (4.6)
**Essential worker, n (%)§**								
No	1452 (73.9)	720 (73.1)	730 (74.8)	766 (77.6)	772 (78.9)	606 (62.3)	676 (71.0)	729 (74.5)
Yes: health	176 (8.9)	90 (9.2)	78 (8.0)	60 (6.1)	97 (9.9)	107 (11.0)	104 (11.0)	87 (8.9)
Yes: non-health	338 (17.2)	174 (17.7)	168 (17.2)	161 (16.3)	111 (11.3)	260 (26.7)	171 (18.0)	162 (16.5)

USA – United States of America, NZ – New Zealand, SD – standard deviation

†28 participants declared a gender identity that was neither female nor male and a further 14 participants preferred not to answer the question; these 42 (0.5%) participants had their gender set to missing.

‡Includes those living with grandparent(s), sibling(s), child(ren) without partner, and those living with family and non-family members.

§Includes 61 participants who responded that they did not know or preferred not to answer.

‖234 (2.6%) participants were unsure or declined to answer and their values were set to missing.

While we found no difference in sex (*P* = 0.79) between countries, we observed a statistically significant difference in age (*P* < 0.001), usual household composition (*P* < 0.001), and essential worker (*P* < 0.001) distributions. Reflecting cultural differences, participants from the Philippines had a younger average age and were more likely to live with extended family, and Hong Kong participants were more likely to be non-health essential workers (**Table 1**).

### Trust

**Figure 1:**
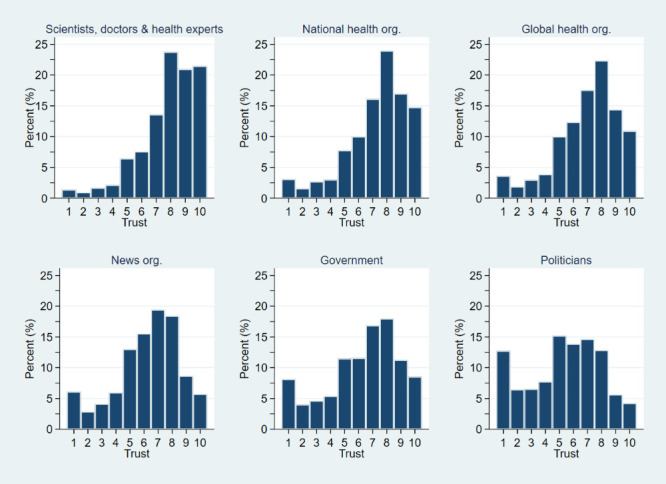
Histograms of respondents’ reported level of trust in experts and organisations (org.). The trust scale ranges from 1 (very low) to 10 (very high).

Regarding the six individual questions, we observed higher levels of trust towards all groups, except for politicians. However, a proportion of participants reported very low levels of trust for each domain (**Figure 1**).

Trust in health experts ranked highest overall and in each of the eight countries, while trust in politicians ranked the lowest overall and in each country. Trust in national health organisations ranked second (except in Hong Kong, where participants ranking trust in news organisations second), and trust in global health organisations ranked third (except in Switzerland and New Zealand, where participants both ranked trust in government third) (**Table 2**).

**Table 2:** Mean and standard error for the six reported levels of trust variables – overall and partitioned by country

Level of trust, x̄ (SE)	Overall	Canada	USA	England	Belgium	Switzerland	Hong Kong	Philippines	NZ
Health experts	7.83 (0.02)*	8.09 (0.06)*	7.80 (0.08)*	7.61 (0.06) *	7.65 (0.06) *	7.25 (0.07) *	7.70 (0.06) *	8.24 (0.07) *	8.07 (0.06) *
National health org.	7.30 (0.03)†	7.65 (0.07)†	7.05 (0.08)†	7.59 (0.06)†	7.07 (0.07)†	6.91 (0.07)†	6.37 (0.08)§	7.97 (0.07)†	7.81 (0.06)†
Global health org.	6.97 (0.03)‡	7.19 (0.07)‡	6.83 (0.09)‡	7.03 (0.07)‡	6.69 (0.06)‡	6.35 (0.07)§	6.54 (0.08)‡	7.63 (0.08)‡	6.90 (0.07)§
Government	6.28 (0.03)§	6.70 (0.07)§	5.28 (0.09)‖	5.86 (0.08)‖	5.42 (0.08)‖	6.44 (0.08)‡	5.59 (0.10)‖	7.14 (0.09)‖	7.37 (0.08)‡
News org.	6.26 (0.03)‖	6.27 (0.07)‖	5.78 (0.10)‡	6.14 (0.07)‡	5.92 (0.07)‡	5.92 (0.07)‖	6.56 (0.06)†	7.17 (0.08)‡	6.30 (0.07)‖
Politicians	5.34 (0.03)¶	5.68 (0.08)¶	4.32 (0.09)¶	5.13 (0.09)¶	4.84 (0.08)¶	5.54 (0.08)¶	5.13 (0.09)¶	5.61 (0.10)¶	6.18 (0.08)¶

x̄ – mean, SE – standard error, USA – United States of America, NZ – New Zealand

*Rank 1.

†Rank 2.

‡Rank 3.

§Rank 4.

‖Rank 5.

¶Rank 6.

We observed significant differences in the mean level of trust between countries for each of the six separate variables (all *P* < 0.001). For instance, although ranked lowest in both countries, trust in politicians for New Zealand participants was higher than in the USA (x̄ = 6.18 vs. x̄ = 4.32. When comparing overall ranked means between variables, they were all significantly different (all *P* < 0.001) except for between government (x̄ = 6.28) and news organisations (mean = 6.26; *P* = 0.45).

### Political orientation

With Hong Kong participants excluded, we obtained valid responses on political orientation from 8026 participants, with 281 (3.5%) declaring as far left, 553 (6.9%) as far right, and 3427 (42.7%) as moderate (**Table 3**).

**Table 3:** Participants’ political orientation to their trust in health authorities, government/politicians, and the news media*

		Trust in health authorities		Trust in government/politicians		Trust in news media	
**Political orientation**	**n (%)**	**Crude x̄ (95% CI)†**	**Adjusted x̄ (95% CI)‡**	**Crude x̄ (95% CI)†**	**Adjusted x̄ (95% CI)‡**	**Crude x̄ (95% CI)†**	**Adjusted x̄ (95% CI)‡**
0 (Far left)	281 (3.5)	0.53 (0.25, 0.81)	0.66 (0.40, 0.93)	-0.38 (-0.73, -0.02)	-0.04 (-0.41, 0.33)	0.19 (-0.17, 0.55)	0.38 (0.02, 0.75)
1	511 (6.4)	0.75 (0.57, 0.93)	0.75 (0.57, 0.92)	0.07 (-0.16, 0.29)	0.12 (-0.12, 0.35)	0.52 (0.31, 0.74)	0.57 (0.36, 0.79)
2	966 (12.0)	0.52 (0.37, 0.66)	0.44 (0.30, 0.59)	0.08 (-0.10, 0.26)	0.05 (-0.12, 0.23)	0.40 (0.23, 0.57)	0.35 (0.18, 0.52)
3 (Moderate)	3427 (42.7)	0 (reference)	0 (reference)	0 (reference)	0 (reference)	0 (reference)	0 (reference)
4	1386 (17.3)	-0.17 (-0.31, -0.02)	-0.18 (-0.31, -0.04)	0.10 (-0.08, 0.27)	0.05 (-0.12, 0.21)	-0.20 (-0.37, -0.02)	-0.20 (-0.37, -0.04)
5	902 (11.2)	-0.02 (-0.19, 0.14)	0.01 (-0.16, 0.17)	0.49 (0.28, 0.70)	0.45 (0.24, 0.67)	0.21 (0.01, 0.42)	0.23 (0.02, 0.44)
6 (Far right)	553 (6.9)	0.24 (-0.01, 0.49)	0.31 (0.05, 0.57)	0.88 (0.57, 1.19)	0.94 (0.62, 1.26)	0.23 (-0.08, 0.55)	0.25 (-0.07, 0.58)

x̄ – mean, CI – confidence interval

*Political orientation data unavailable from Hong Kong participants.

†Adjusted for country.

‡Adjusted for gender, age group, household composition, work-force status, perceived threat to self, perceived threat to country, financial losses, stress, and country.

### Information sources

For the combined information sources domain scores, complete non-missing responses ranged from 92.0% (n = 8290) for the derived social media variable to 96.0% (n = 8688) for health. Overall, participants sourced information to a higher degree (i.e. responses in the mainly/always or often range) from health (n = 4664 (53.7%)), government (n = 3448 (40.0%)), news media (n = 3791 (43.9%)), and social media (n = 2694 (32.5%)) sources (**Table 4**). Participant correlations between these information sources was modest, ranging from *R* = 0.20 (government and social media sources) to *R* = 0.39 (health and government sources).

**Table 4:** Participants’ information source level to their trust in health authorities, government/politicians, and the news media

		Trust in health authorities		Trust in government/politicians		Trust in news media	
**Information source**	**n (%)**	Crude x̄ (95% CI)*	**Adjusted x̄ (95% CI)†**	Crude x̄ (95% CI)*	**Adjusted x̄ (95% CI)†**	Crude x̄ (95% CI)*	**Adjusted x̄ (95% CI)†**
Health							
*Higher*	4664 (53.7)	0 (reference)	0 (reference)	0 (reference)	0 (reference)	0 (reference)	0 (reference)
*Lower*	4024 (46.3)	-1.12 (-1.22, -1.02)	-1.04 (-1.14, -0.94)	-0.51 (-0.64, -0.39)	-0.50 (-0.63, -0.38)	-0.89 (-1.01, -0.77)	-0.82 (-0.94, -0.70)
Government							
*Higher*	3448 (40.0)	0 (reference)	0 (reference)	0 (reference)	0 (reference)	0 (reference)	0 (reference)
*Lower*	5180 (60.0)	-0.59 (-0.68, -0.50)	-0.58 (-0.68, -0.49)	-1.55 (-1.67, -1.43)	-1.52 (-1.64, -1.40)	-0.64 (-0.75, -0.52)	-0.61 (-0.73, -0.50)
News media							
*Higher*	3791 (43.9)	0 (reference)	0 (reference)	0 (reference)	0 (reference)	0 (reference)	0 (reference)
*Lower*	4841 (56.1)	-0.41 (-0.50, -0.31)	-0.35 (-0.44, -0.26)	-0.54 (-0.66, -0.42)	-0.50 (-0.62, -0.38)	-1.17 (-1.28, -1.06)	-1.12 (-1.24, -1.01)
Social media							
*Higher*	2694 (32.5)	0 (reference)	0 (reference)	0 (reference)	0 (reference)	0 (reference)	0 (reference)
*Lower*	5596 (6.75)	0.22 (0.11, 0.33)	0.06 (-0.05, 0.17)	-0.11 (-0.25, 0.03)	-0.25 (-0.39, -0.10)	-0.07 (-0.19, 0.06)	-0.17 (-0.30, -0.04)

x̄ – mean, CI – confidence interval

*Adjusted for country.

†Adjusted for gender, age group, household composition, work-force status, perceived threat to self, perceived threat to country, financial losses, stress, and country.

### Crude analyses

Although trust was measured by six variables, the correlation between some variables was strong. Trust in health authorities or experts (questions 1-3) had correlations ranging between 0.71-0.81 (Table S1 in the **Online Supplementary Document**). Similarly, trust in government/politicians (questions 4-5) had a correlation of 0.83. To reduce interdependence and test multiplicity, we derived and adopted three primary measures: trust in health authorities, which combined responses to questions 1-3, trust in government/politicians, which combined responses to questions 4-5, and trust in news organisations, which was given by question 6 for these regression analyses. Initially, we considered conducting a principal component analysis to create the combined scores, but the first components for each were almost linear combinations of the apposite variables, so we opted to simply sum the response scores. As the number of component variables were different for each derived primary trust variable, we re-scaled these summed scored to lie on the original 10-point scale, thereby enabling direct comparisons and facilitating interpretation.

In the six separate regression analyses of political orientation and information sources against the three composite trust variables, moderate political orientation and higher information source levels were taken as reference categories.

**Figure 2:**
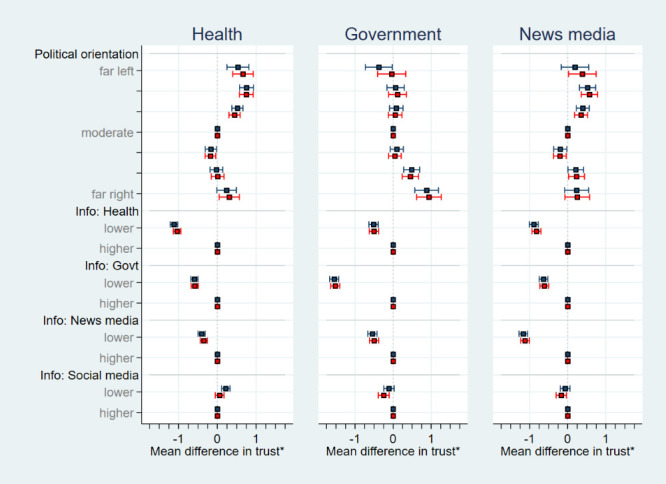
Mean differences in level of trust, together with associated 95% CIs, derived from crude linear regression analyses (adjusted for country; denoted by blue square) and adjusted linear regression analyses (adjusted for gender, age group, household composition, work-force status, perceived threat to self, perceived threat to country, financial losses, stress, and country; denoted by red squares) relating participants’ political orientation and information source level to their trust in health authorities, government/politicians, and the news media. *A positive mean difference indicates a higher level of trust compared to the reference group, whereas a negative mean difference indicates a relatively lower level of trust. CI – confidence interval, Info – information source.

Participants oriented towards the left had a higher trust in health authorities and, to a lesser extent, in the news media than their right leaning counterparts, while those oriented towards the right had a relatively higher trust in government and politicians (**Figure 2**). Additionally, participants using higher levels of health information sources had the highest trust in health authorities, while those using higher levels of government information sources and higher levels of new media information sources had the highest trust in government/politicians and news media, respectively (**Figure 2**). However, there was little difference in trust in health, government or news media by those using higher or lower levels of social media information sources. Nonetheless, all these reported associations were significant (*P* < 0.001) (**Table 3**, **Table 4**, and Tables S2 and S3 in the **Online Supplementary Document**.

### Adjusted analyses

While we observed some confounding in the adjusted regression analyses, the patterns described for the crude analyses remain largely unaltered. All the reported associations were significant (*P* < 0.001) apart from social media information source level related to trust in news media (*P* = 0.01) and social media information source level related to trust in health authorities (*P* = 0.30) (**Figure 2**, **Table 3**, **Table 4**, and Tables S2 and S3 in the **Online Supplementary Document**).

## DISCUSSION

Trust in health experts ranked highest across all eight participating countries, while conversely, trust in politicians ranked lowest. Unlike the ranking for health experts, there was considerable heterogeneity in the mean scores for trust in politicians between countries. At the extremes, trust in politicians was the lowest in the USA (x̄ = 4.32) and the highest in New Zealand’s (x̄ = 6.18) (on a 10-point scale ranging from 1 (very low) to10 (very high)). At the beginning of the pandemic, USA and New Zealand had very different approaches to health messaging from their respective leaders, with New Zealand’s prime minister at that time globally heralded for her leadership in the nation’s COVID-19 response [25]. Messaging was swift, informed, and cohesive with the information provided by the nation’s Ministry of Health. By comparison, the USA president released conflicting information to his chief medical advisor, creating confusion and distrust to medical advice and poor agenda management [25]. Notably, as of 3 May 2023, USA’s total COVID-19 attributable death rate per 100 000 was estimated at 339.59, approximately 6.0 times higher than New Zealand’s rate of 56.74 [1].

Another known element in the trust of governments and politicians is people’s perceptions of corruption and non-transparency [26]. The Corruption Perceptions Index (CPI) measures corruption within the public sectors globally. For 2020, the time of this study, USA and the Philippines ranked the lowest among the eight participating countries, while New Zealand and Switzerland ranked highest [26]. The heterogeneity in the mean scores for trust in politicians between countries seen in this study may reflect these CPI differences. Hong Kong’s CPI was ranked third from the eight countries, yet we observed a notable distrust in politicians. This may reflect lingering ill-feelings arising from the 2019 protests concerning the One Country-Two Systems model, under which Hong Kong and Beijing currently operate, and a growing unease of the pervasiveness of China as a “second government” in Hong Kong affairs [20]. Despite these political differences and events eroding political trust, its impact on Hong Kong people’s trust in health experts appeared largely unaffected. The mean values for trust in health experts and authorities for USA and Hong Kong participants was only slightly below the all-country mean, and was highest among Philippines participants. Thus, entrusting and empowering health authorities and experts to provide (and to be seen providing) coordinated accurate health messaging, information, and recommendations is likely a fundamental strategy in ensuring the highest likelihood of population compliance [8,9,25]. Coordination is also key, as political trust is instrumental for compliance with health measures [5,27,28].

Individuals’ political orientation and political alignment is also an important indicator for compliance with public health measures [11,12]. We found that left-oriented individuals tended to have higher trust in both health authorities and news media, whereas those who were right-oriented had more trust in government and politicians. Differing political ideologies have been shown to affect the perceived impact of COVID-19 prevention behaviours [12]. Generally, people with conservative ideologies emphasise on personal responsibility and self-autonomy. By comparison, those with liberal ideologies prioritise the collective and the interdependence between different groups. Political ideology has transformed the public response to health mandates – something as simple as choosing to wear or not wear a mask could become a symbolic expression of political support [27]. Having such divisive and politicised public responses to health mandates can undermine the positive effect of timely collective action on slowing the rate of infection and protecting the most vulnerable members of the community. Importantly, our findings must be contextualised, as most of the eight countries studied here held right-wing offices at the time of the study. Partisan alignment and government approval are strongly linked; an individual’s alignment with party in office is a predictor of governmental approval [28], and may in turn allow for the implementation of impactful health policies to address domestic concerns – for example, vaccine mandates and lockdown procedures [5]. Future research should investigate the interaction between federal, regional, and individual political orientations and their alignment or misalignment, and how this might affect individual’s trust, confidence and sense of coherence.

The COVID-19 infodemic has highlighted the effect of misinformation, disinformation, and/or conflicting information on causing confusion and risk-taking behaviours that can harm health. It also leads to mistrust in health authorities and undermines the public health response [17]. Our analyses demonstrated that, in almost all instances (except for social media), the use of lower levels of information sources had a significant association with reduced trust. Discourse on disinformation and conspiracy theories preys on people’s fears and vulnerability during a period of uncertainty, and provides simple, yet often false explanations regarding complex and fraught situations [16]. Many of the emerging narratives regarding COVID-19 have health messaging that conflicts with that given by health experts, such as alternative cures for COVID-19, and “debunking” the efficacy of vaccines and mask-use [29]. As collective action is key to pandemic response, misleading information campaigns threaten the health and well-being of society. The relatively small effect size difference in trust between higher and lower social media user groups observed here likely points to a lack of nuance. Many users (higher or otherwise) engaged in social media for many different purposes and may be unfamiliar with much of the malevolent content. In retrospect, a better question might have focused on the nature and source of the social media content rather than simply the frequency of use.

This study has several strengths, such as its relatively large sample size, and the timeliness and global spread of participant recruitment across eight countries in four continents. However, it also has important limitations. First, the survey was conducted during the COVID-19 pandemic, and thus lacks baseline or pre-pandemic data, which also may distort the presented associations and findings. Replication post-pandemic would serve to understand the stability or otherwise of these relationships. Moreover, unmeasurable non-sampling bias is also an important limitation. While participant selection was stratified and randomised, the sampling frame included online and offline sources, which are less transparent than traditional methodologies and likely reduce the coverage for some subpopulations (such as those with mental or physical health disabilities [30]). However, we used targeted quota sampling and survey weights to mitigate these issues and ensure approximately representative samples. Another important limitation pertains to the primary variables themselves, including the elicited level of trust. While having good face validity, and being informed by guidelines [19] and previous studies, these variables have no published psychometric properties and may suffer from responder bias, and they may have suboptimal test content validity and reliability. Further work investigating the variable psychometrics is vital. Additionally, instrument translation and validation processes associated with the instruments were undertaken within the research team. There were numerous pragmatic considerations and concessions made in designing, attracting funding, securing ethics, and implementing this international study within a relatively short time frame [21,22]. One internal requirement was that at least one national lead for each included country was deemed necessary, to ensure cultural and contextual guidance and appropriateness. Leads were also responsible for ensuring the instrument translations were linguistically sound. However, limited back-translating and validation processes were undertaken, so some country-specific differences may have been the results of language subtleties. Another potential weakness is the cross-sectional rather than longitudinal study design, which negates any causal assertions. Here, the directional and bi-directional nature of the trust relationships between the public, experts, and organisations cannot be distinguished. The political responses and health policies to the pandemic evolve swiftly over a short period of time, and people’s feelings regarding the pandemic similarly change. Therefore, our findings are representative of populations in the relatively early stages of the pandemic and may differ if this study was repeated later. Finally, unmeasured confounders are another source of potential error, and may affect the presented comparative analyses between countries. Such confounders can lead to substantial bias in the estimated exposure-outcome estimates, particularly if they are uncorrelated with the considered explanatory variables [31]. Study replication using different suites of variables is needed to understand their effect.

## CONCLUSIONS

Trust is a key determinant of health; it is central to the successful implementation and uptake of public health measures. Although it shifts easily and significantly impacts society, it has received relatively little public health research attention. This study empirically highlights the universally high levels of public trust placed in health experts and authorities across eight countries. We recommend that governments and policymaker coordinate their response with health experts and authorities, thereby maximising the likely population health impact. COVID-19 is not the first, and certainly will not be the last, global health crisis that we have faced. However, it is the first to exist on this scale during the “infodemic” in the new digital landscape of the internet and media. We need to learn the lessons from COVID-19 to inform successful response for future health crises and challenges.

## Additional material


Online Supplementary Document
