# Supplementary material for: In the COVID-19 pandemic, who did we trust? An eight-country cross-sectional study
Source: J Glob Health. 2023 Sep 1;13:06036. doi: 10.7189/jogh.13.06036 (PMC10471152; doi:10.7189/jogh.13.06036)
Supplement: Online Supplementary Document [file jogh-13-06036-s001.pdf]

APPENDIX

Table S1. Correlation matrix between respondents' level of trust in experts and organisations (org.) variables

	Health experts	National health org	Global health org	Government	Politicians	News org.
Health experts	1					
National health org.	0.75	1				
Global health org.	0.71	0.81	1			
Government	0.52	0.68	0.61	1		
Politicians	0.43	0.58	0.55	0.83	1	
News org.	0.59	0.61	0.67	0.60	0.63	1.00

Table S2. Mean differences together with associated 95% confidence intervals (CIs) derived from crude and adjusted regression analyses relating participants' political orientation to their trust in health authorities, government/politicians, and the news media

			**Trust in health authorities**		**Trust in government/politicians**		**Trust in the news media**	
			**Crude***	**Adjusted†**	**Crude***	**Adjusted†**	**Crude***	**Adjusted†**
	**n**	**(%)**	**mean (95% CI)**	**mean (95% CI)**	**mean (95% CI)**	**mean (95% CI)**	**mean (95% CI)**	**mean (95% CI)**
**Political orientation**								
0 (Far left)	281	(3.5)	0.53 (0.25, 0.81)	0.66 (0.40, 0.93)	-0.38 (-0.73, -0.02)	-0.04 (-0.41, 0.33)	0.19 (-0.17, 0.55)	0.38 (0.02, 0.75)
1	511	(6.4)	0.75 (0.57, 0.93)	0.75 (0.57, 0.92)	0.07 (-0.16, 0.29)	0.12 (-0.12, 0.35)	0.52 (0.31, 0.74)	0.57 (0.36, 0.79)
2	966	(12.0)	0.52 (0.37, 0.66)	0.44 (0.30, 0.59)	0.08 (-0.10, 0.26)	0.05 (-0.12, 0.23)	0.40 (0.23, 0.57)	0.35 (0.18, 0.52)
3 (Moderate)	3,427	(42.7)	0 (reference)	0 (reference)	0 (reference)	0 (reference)	0 (reference)	0 (reference)
4	1,386	(17.3)	-0.17 (-0.31, -0.02)	-0.18 (-0.31, -0.04)	0.10 (-0.08, 0.27)	0.05 (-0.12, 0.21)	-0.20 (-0.37, -0.02)	-0.20 (-0.37, -0.04)
5	902	(11.2)	-0.02 (-0.19, 0.14)	0.01 (-0.16, 0.17)	0.49 (0.28, 0.70)	0.45 (0.24, 0.67)	0.21 (0.01, 0.42)	0.23 (0.02, 0.44)
6 (Far right)	553	(6.9)	0.24 (-0.01, 0.49)	0.31 (0.05, 0.57)	0.88 (0.57, 1.19)	0.94 (0.62, 1.26)	0.23 (-0.08, 0.55)	0.25 (-0.07, 0.58)
**Gender**								
Females	4,117	(51.6)		0.03 (-0.07, 0.12)		0.09 (-0.04, 0.21)		-0.01 (-0.13, 0.10)

Males	3,867	(48.4)	0 (reference)	0 (reference)	0 (reference)
**Age (years)**					
18-24	900	(11.2)	0 (reference)	0 (reference)	0 (reference)
25-34	1,484	(18.5)	-0.02 (-0.21, 0.17)	0.11 (-0.13, 0.35)	0.13 (-0.09, 0.36)
35-44	1,287	(16.0)	0.05 (-0.15, 0.25)	0.12 (-0.13, 0.36)	0.28 (0.04, 0.52)
45-54	1,465	(18.3)	-0.16 (-0.36, 0.03)	-0.19 (-0.43, 0.06)	-0.02 (-0.26, 0.22)
55-64	1,290	(16.1)	0.10 (-0.10, 0.30)	0.08 (-0.17, 0.33)	0.27 (0.03, 0.52)
65-74	1,211	(15.1)	0.19 (-0.02, 0.40)	0.23 (-0.03, 0.49)	0.23 (-0.03, 0.48)
≥75	387	(4.8)	0.33 (0.06, 0.59)	0.27 (-0.04, 0.59)	0.29 (-0.02, 0.60)
**Household composition**					
Alone	1,459	(18.2)	0 (reference)	0 (reference)	0 (reference)
Partner only	2,500	(31.2)	0.15 (0.01, 0.29)	0.10 (-0.08, 0.27)	0.02 (-0.15, 0.19)
Partner & child(ren)	1,757	(21.9)	0.25 (0.09, 0.42)	0.36 (0.16, 0.56)	0.20 (0.01, 0.39)
Other family	2,053	(25.6)	0.13 (-0.04, 0.31)	0.20 (-0.01, 0.41)	0.23 (0.02, 0.43)
Non-family	255	(3.2)	0.04 (-0.30, 0.38)	-0.24 (-0.63, 0.14)	-0.52 (-0.92, -0.12)
**Work-force**					
Non-essential	5,841	(74.7)	0 (reference)	0 (reference)	0 (reference)
Essential work: health	692	(8.9)	0.37 (0.21, 0.53)	0.52 (0.30, 0.73)	0.50 (0.29, 0.71)
Essential work: other	1,283	(16.4)	-0.01 (-0.15, 0.14)	0.28 (0.11, 0.46)	0.03 (-0.14, 0.20)
**Perceived threat to self**					
Very low	947	(12.1)	0 (reference)	0 (reference)	0 (reference)
Low	1,663	(21.3)	0.45 (0.23, 0.68)	0.57 (0.31, 0.83)	0.59 (0.33, 0.86)
Moderate	2,955	(37.9)	0.53 (0.31, 0.75)	0.85 (0.59, 1.10)	0.88 (0.62, 1.13)
High	1,385	(17.8)	0.76 (0.52, 0.99)	1.07 (0.79, 1.36)	1.17 (0.89, 1.45)
Very high	844	(10.8)	0.90 (0.62, 1.17)	1.27 (0.94, 1.60)	1.41 (1.08, 1.73)
**Perceived threat to country**					
Very low	259	(3.3)	0 (reference)	0 (reference)	0 (reference)
Low	597	(7.7)	0.72 (0.25, 1.19)	0.49 (-0.06, 1.03)	0.42 (-0.11, 0.95)
Moderate	2,329	(30.0)	1.25 (0.80, 1.70)	0.86 (0.34, 1.38)	0.79 (0.28, 1.29)
High	2,856	(36.8)	1.48 (1.02, 1.93)	0.72 (0.20, 1.24)	0.87 (0.37, 1.38)
Very high	1,710	(22.1)	1.50 (1.03, 1.97)	0.22 (-0.32, 0.76)	0.78 (0.26, 1.30)
**Financial losses**					
None	2,317	(28.9)	0 (reference)	0 (reference)	0 (reference)
Insignificant	2,086	(26.0)	-0.10 (-0.23, 0.03)	0.02 (-0.14, 0.18)	0.05 (-0.11, 0.21)

Significant	1,887	(23.5)		-0.35 (-0.50, -0.21)		-0.31 (-0.48, -0.13)		-0.22 (-0.39, -0.05)
Very significant	695	(8.7)		-0.51 (-0.74, -0.27)		-0.43 (-0.72, -0.15)		-0.24 (-0.52, 0.04)
Unknown	1,041	(13.0)		-0.11 (-0.36, 0.13)		0.31 (0.02, 0.61)		0.38 (0.11, 0.65)
**Stress**								
None	764	(9.7)		0 (reference)		0 (reference)		0 (reference)
Not very	1,743	(22.1)		-0.09 (-0.28, 0.10)		-0.19 (-0.43, 0.05)		-0.04 (-0.29, 0.20)
A bit	2,917	(37.0)		-0.19 (-0.38, -0.01)		-0.30 (-0.53, -0.06)		-0.13 (-0.37, 0.11)
Rather	1,669	(21.1)		-0.39 (-0.59, -0.18)		-0.51 (-0.77, -0.25)		-0.41 (-0.67, -0.15)
Extremely	797	(10.1)		-0.61 (-0.90, -0.33)		-0.76 (-1.09, -0.42)		-0.45 (-0.78, -0.11)
**Country**								
Canada	2,004	(25.0)	0 (reference)	0 (reference)	0 (reference)	0 (reference)	0 (reference)	0 (reference)
USA	1,003	(12.5)	-0.44 (-0.63, -0.25)	-0.35 (-0.54, -0.15)	-1.47 (-1.69, -1.24)	-1.30 (-1.54, -1.07)	-0.52 (-0.76, -0.29)	-0.41 (-0.65, -0.17)
England	1,000	(12.5)	-0.19 (-0.36, -0.02)	-0.24 (-0.41, -0.08)	-0.73 (-0.95, -0.52)	-0.78 (-1.00, -0.56)	-0.10 (-0.30, 0.10)	-0.23 (-0.43, -0.02)
Belgium	1,014	(12.6)	-0.50 (-0.67, -0.33)	-0.58 (-0.80, -0.35)	-1.07 (-1.28, -0.86)	-1.39 (-1.68, -1.10)	-0.34 (-0.54, -0.15)	-0.69 (-0.95, -0.43)
Switzerland	1,000	(12.5)	-0.82 (-1.00, -0.64)	-0.86 (-1.03, -0.70)	-0.23 (-0.43, -0.02)	-0.26 (-0.46, -0.06)	-0.37 (-0.57, -0.16)	-0.40 (-0.60, -0.20)
Philippines	1,003	(12.5)	0.37 (0.18, 0.56)	0.53 (0.33, 0.73)	0.04 (-0.19, 0.27)	0.26 (0.01, 0.51)	0.93 (0.71, 1.14)	0.91 (0.67, 1.16)
New Zealand	1,001	(12.5)	-0.07 (-0.23, 0.10)	0.18 (0.01, 0.35)	0.55 (0.35, 0.76)	0.64 (0.42, 0.85)	0.02 (-0.18, 0.22)	0.19 (-0.02, 0.40)

Note: political orientation data unavailable from Hong Kong participants; *adjusted for country; †adjusted gender, age group, household composition, work-force status, perceived threat to self, perceived threat to country, financial losses, stress, and country.

Table S3. Mean differences together with associated 95% confidence intervals (CIs) derived from crude and adjusted regression analyses relating participants' information source level to their trust in health authorities, government/politicians, and the news media.

			Trust in health authorities		Trust in Government/politicians		Trust in the news media	
			Crude*	Adjusted†	Crude*	Adjusted†	Crude*	Adjusted†
	n	(%)	mean (95% CI)	mean (95% CI)	mean (95% CI)	mean (95% CI)	mean (95% CI)	mean (95% CI)
**Health information source**								
Higher	4,664	(53.7)	0 (reference)	0 (reference)	0 (reference)	0 (reference)	0 (reference)	0 (reference)
Lower	4,024	(46.3)	-1.12 (-1.22, -1.02)	-1.04 (-1.14, -0.94)	-0.51 (-0.64, -0.39)	-0.50 (-0.63, -0.38)	-0.89 (-1.01, -0.77)	-0.82 (-0.94, -0.70)
**Government information source**								
Higher	3,448	(40.0)	0 (reference)	0 (reference)	0 (reference)	0 (reference)	0 (reference)	0 (reference)
Lower	5,180	(60.0)	-0.59 (-0.68, -0.50)	-0.58 (-0.68, -0.49)	-1.55 (-1.67, -1.43)	-1.52 (-1.64, -1.40)	-0.64 (-0.75, -0.52)	-0.61 (-0.73, -0.50)
**News media information source**								

Higher	3,791	(43.9)	0 (reference)	0 (reference)	0 (reference)	0 (reference)	0 (reference)	0 (reference)
Lower	4,841	(56.1)	-0.41 (-0.50, -0.31)	-0.35 (-0.44, -0.26)	-0.54 (-0.66, -0.42)	-0.50 (-0.62, -0.38)	-1.17 (-1.28, -1.06)	-1.12 (-1.24, -1.01)
**Social media information source**								
Higher	2,694	(32.5)	0 (reference)	0 (reference)	0 (reference)	0 (reference)	0 (reference)	0 (reference)
Lower	5,596	(67.5)	0.22 (0.11, 0.33)	0.06 (-0.05, 0.17)	-0.11 (-0.25, 0.03)	-0.25 (-0.39, -0.10)	-0.07 (-0.19, 0.06)	-0.17 (-0.30, -0.04)
**Gender**								
Females	4,667	(51.9)		0.03 (-0.06, 0.11)		0.08 (-0.03, 0.19)		0.03 (-0.07, 0.14)
Males	4,318	(48.1)		0 (reference)		0 (reference)		0 (reference)
**Age (years)**								
18-24	995	(11.0)		0 (reference)		0 (reference)		0 (reference)
25-34	1,657	(18.4)		-0.05 (-0.23, 0.12)		0.11 (-0.10, 0.33)		0.06 (-0.14, 0.27)
35-44	1,468	(16.3)		0.01 (-0.18, 0.20)		0.16 (-0.07, 0.39)		0.21 (-0.01, 0.43)
45-54	1,657	(18.4)		-0.06 (-0.24, 0.13)		0.06 (-0.17, 0.29)		0.01 (-0.21, 0.23)
55-64	1,467	(16.3)		0.10 (-0.09, 0.29)		0.16 (-0.07, 0.39)		0.20 (-0.03, 0.42)
65-74	1,382	(15.3)		0.02 (-0.18, 0.22)		0.23 (-0.02, 0.48)		0.03 (-0.21, 0.27)
≥75	401	(4.4)		0.18 (-0.07, 0.43)		0.38 (0.08, 0.68)		0.07 (-0.24, 0.38)
**Household composition**								
Alone	1,527	(16.9)		0 (reference)		0 (reference)		0 (reference)
Partner only	2,680	(29.7)		0.04 (-0.09, 0.18)		0.02 (-0.15, 0.19)		-0.12 (-0.28, 0.04)
Partner & child(ren)	2,069	(22.9)		0.18 (0.02, 0.33)		0.32 (0.13, 0.51)		0.08 (-0.10, 0.26)
Other family	2,488	(27.6)		0.07 (-0.09, 0.23)		0.18 (-0.02, 0.37)		0.11 (-0.07, 0.30)
Non-family	264	(2.9)		0.09 (-0.24, 0.43)		-0.07 (-0.43, 0.29)		-0.49 (-0.89, -0.09)
**Work-force**								
Non-essential	6,450	(73.3)		0 (reference)		0 (reference)		0 (reference)
Essential work: health	800	(9.1)		0.13 (-0.01, 0.27)		0.32 (0.12, 0.51)		0.23 (0.05, 0.42)
Essential work: other	1,544	(17.6)		-0.10 (-0.23, 0.02)		0.15 (-0.01, 0.31)		-0.08 (-0.22, 0.07)
**Perceived threat to self**								
Very low	1,024	(11.7)		0 (reference)		0 (reference)		0 (reference)
Low	1,880	(21.4)		0.42 (0.20, 0.64)		0.52 (0.28, 0.76)		0.59 (0.35, 0.84)
Moderate	3,347	(38.1)		0.36 (0.14, 0.58)		0.64 (0.40, 0.88)		0.73 (0.49, 0.97)
High	1,567	(17.8)		0.49 (0.26, 0.73)		0.78 (0.51, 1.04)		0.85 (0.58, 1.11)
Very high	970	(11.0)		0.47 (0.21, 0.73)		0.80 (0.50, 1.10)		0.94 (0.65, 1.24)
**Perceived threat to country**								
Very low	296	(3.4)		0 (reference)		0 (reference)		0 (reference)

Low	721	(8.2)		0.67 (0.27, 1.08)		0.33 (-0.12, 0.77)		0.37 (-0.05, 0.78)
Moderate	2,676	(30.6)		1.00 (0.61, 1.39)		0.57 (0.15, 1.00)		0.69 (0.29, 1.09)
High	3,195	(36.5)		1.10 (0.71, 1.50)		0.33 (-0.10, 0.76)		0.71 (0.31, 1.11)
Very high	1,860	(21.3)		1.17 (0.77, 1.57)		-0.13 (-0.57, 0.32)		0.57 (0.16, 0.99)
**Financial losses**								
None	2,494	(27.6)		0 (reference)		0 (reference)		0 (reference)
Insignificant	2,485	(27.5)		-0.20 (-0.32, -0.08)		-0.05 (-0.20, 0.10)		-0.07 (-0.21, 0.07)
Significant	2,175	(24.1)		-0.33 (-0.46, -0.19)		-0.25 (-0.42, -0.09)		-0.23 (-0.38, -0.07)
Very significant	804	(8.9)		-0.43 (-0.63, -0.23)		-0.29 (-0.55, -0.04)		-0.20 (-0.43, 0.03)
Unknown	1,069	(11.8)		-0.16 (-0.41, 0.09)		0.22 (-0.07, 0.51)		0.27 (0.01, 0.53)
**Stress**								
None	805	(9.1)		0 (reference)		0 (reference)		0 (reference)
Not very	1,903	(21.4)		0.05 (-0.14, 0.23)		-0.05 (-0.28, 0.17)		0.02 (-0.20, 0.24)
A bit	3,379	(38.0)		-0.12 (-0.30, 0.07)		-0.21 (-0.43, 0.01)		-0.15 (-0.37, 0.07)
Rather	1,874	(21.1)		-0.23 (-0.44, -0.03)		-0.40 (-0.65, -0.16)		-0.28 (-0.52, -0.05)
Extremely	922	(10.4)		-0.39 (-0.64, -0.13)		-0.65 (-0.96, -0.35)		-0.28 (-0.58, 0.02)
**Country**								
Canada	2,004	(22.2)	0 (reference)	0 (reference)	0 (reference)	0 (reference)	0 (reference)	0 (reference)
USA	1,003	(11.1)	-0.26 (-0.44, -0.08)	-0.19 (-0.37, -0.01)	-1.10 (-1.31, -0.88)	-0.94 (-1.16, -0.72)	-0.35 (-0.57, -0.12)	-0.25 (-0.48, -0.02)
England	1,000	(11.1)	-0.12 (-0.29, 0.04)	-0.16 (-0.32, 0.01)	-0.60 (-0.81, -0.38)	-0.60 (-0.82, -0.39)	-0.10 (-0.30, 0.10)	-0.20 (-0.40, -0.01)
Belgium	1,014	(11.2)	-0.53 (-0.70, -0.36)	-0.50 (-0.74, -0.26)	-1.02 (-1.23, -0.81)	-1.21 (-1.50, -0.92)	-0.49 (-0.69, -0.30)	-0.74 (-1.00, -0.48)
Switzerland	1,000	(11.1)	-0.81 (-0.98, -0.64)	-0.82 (-0.98, -0.65)	-0.27 (-0.46, -0.07)	-0.27 (-0.47, -0.07)	-0.49 (-0.68, -0.29)	-0.50 (-0.69, -0.30)
Hong Kong	1,002	(11.1)	-0.71 (-0.88, -0.55)	-0.64 (-0.81, -0.46)	-0.75 (-0.96, -0.53)	-0.84 (-1.06, -0.61)	0.16 (-0.03, 0.35)	0.12 (-0.08, 0.32)
Philippines	1,003	(11.1)	-0.02 (-0.20, 0.16)	0.10 (-0.09, 0.29)	-0.27 (-0.48, -0.05)	-0.08 (-0.31, 0.16)	0.24 (0.03, 0.45)	0.23 (-0.01, 0.46)
New Zealand	1,001	(11.1)	-0.08 (-0.24, 0.07)	0.07 (-0.09, 0.23)	0.57 (0.38, 0.76)	0.57 (0.37, 0.77)	-0.05 (-0.24, 0.14)	0.07 (-0.13, 0.27)

Note: *adjusted for country; †adjusted for gender, age group, household composition, work-force status, perceived threat to self, perceived threat to country, financial losses, stress, and country.
